# Responses of sap flow density of two shrub species to rainfall classes on the semiarid Loess Plateau of China

**DOI:** 10.3389/fpls.2023.1237248

**Published:** 2023-08-11

**Authors:** Weiwei Fang, Nan Lu, Jianbo Liu, Ruiping Li, Yuxiao Wang

**Affiliations:** ^1^ Key Research Institute of Yellow Civilization and Sustainable Development & Collaborative Innovation Center of Henan Province, Henan University, Kaifeng, China; ^2^ State Key Laboratory of Urban and Regional Ecology, Research Center for Eco-Environmental Sciences, Chinese Academy of Sciences, Beijing, China; ^3^ National Observation and Research Station of Earth Critical Zone on the Loess Plateau in Shaanxi, Xi’an, China; ^4^ University of Chinese Academy of Sciences, Beijing, China; ^5^ Tianjin Key Laboratory of Water Resources and Environment, Tianjin Normal University, Tianjin, China

**Keywords:** sap flow density, rainfall event, leaf water potential, semiarid area, Loess Plateau

## Abstract

**Introduction:**

Rainfall events can determine a cascade of plant physiological and ecological processes, and there is considerable interest in the way that rainfall modifies plant water flux dynamics.

**Methods:**

The sap flow density (SF) of the planted species of *Vitex negundo* and *Hippophae rhamnoides*, on the Loess Plateau of China was monitored using the heat balance method from 2015 to 2017.

**Results and discussion:**

The results showed that SF responded differently to rainfall classes because of the changing meteorological and soil water content (SWC) conditions. For class 1: 0.2–2 mm, SF increased by 14.36–42.93% for the two species, which were mainly attributable to the effect of solar radiation and vapor pressure deficit after rainfall. For class 2: 2–10 mm, SF remained nearly stable for *V. negundo* and decreased for *H. rhamnoides* because of the relative humidity’s effect. For class 3: > 10 mm, SF increased significantly because of increased SWC and the increasing response to solar radiation. The increased percentage of SF was relatively higher for *V. negundo* when rainfall was less than 20 mm, while the value was higher for *H. rhamnoides* when rainfall was greater than 10 mm. Further, *V. negundo*’s water potential increased at the soil–root interface (ψ_0_) and ψ_L_, indicating that the plant, which has shallower roots and a coarser of leaf and bark texture, considered as anisohydric species and used precipitation-derived upper soil water to survive. The relatively consistent ψ_L_ and ψ_0_ for *H. rhamnoides*, which has deep roots and leathery leaves, indicated that this species was considered as isohydric species and insensitive to the slight change in the soil water status. The differed response patter and water use strategies between the two species showed that species as *V. negundo* are more susceptible to frequent, but small rainfall events, while larger, but less frequent rainfall events benefit such species as *H. rhamnoides*. This study quantified the effect of environmental factors for SF variation. The results could help formulate a selection process to determine which species are more suitable for sustainable management in the afforestation activities under the context of more frequent and intense rainfall events.

## 1 Introduction

Rainfall events in arid and semiarid regions are discontinuous, highly variable, and largely unpredictable with respect to frequency and intensity in total rainfall amount, and they are key determinants of ecological and plant physiological processes (Sun et al., 2015; Dietrich and Kahmen, 2019; Iqbal et al., 2021). Further, global climate change appears to increase rainfall patterns’ variability, which would has a more significant influence on vegetation’s survival and water use than its annual and seasonal amount (Hao et al., 2023; Ni et al., 2023). Accordingly, research has focused on discontinuous rainfall events of varied amounts rather than seasonal or annual precipitation (Huang and Zhang, 2016; Iqbal et al., 2021). Understanding vegetation’s physiological responses to soil moisture and atmospheric drought after rainfall and their underlying mechanisms are critical to predict plant’s water use accurately and their future changes in response to the shifts in rainfall patterns in arid and semi-arid areas.

Sap flow density (SF) is an indicator of plant transpiration that can reflect the individual plant’s physiological characteristics and water use response to environmental factors (Wu et al., 2018; Liu et al., 2022). In addition to meteorological factors and vegetation conditions, the variation of SF has been shown to be related to soil water availability, which is strongly regulated by precipitation, particularly in water limited regions (Wang et al., 2017a; Mu et al., 2022). This regulation may be related to multiple factors, such as rainfall characteristics (Zeppel et al., 2008; Huang and Zhang, 2016; Wang et al., 2020), meteorological factors after rainfall, and habitat. Rainfall influences plant water fluxes from the root zone to the atmosphere via leaf stomata by influencing soil water and meteorological factors, and their relative contributions vary under different rainfall events. Many site-level studies have found significant and positive correlations between soil water content (SWC) and SF (Kume et al., 2007; Chang et al., 2014). When rainfall events were too small to affect soil moisture, the changing meteorological conditions became SF’s main determinants SF in response to rainfall events (Huang and Zhang, 2016; Wang et al., 2017a). Therefore, because of different determining factors, SF’s the response time lag also differs depending upon rainfall amounts.

In addition to abiotic factors, biological factors, such as plant morphology (e.g., root depth and leaf roughness) and physiological properties, affect SF’s responses to rainfall events (Zeppel et al., 2008; Huang and Zhang, 2016), for example, the SF of shallow-rooted species such as *Haloxylon ammodendron* (C.A.Mey.) Bunge used rainfall-derived upper soil water for survival, while the deep-rooted species *Tamarix ramosissima* Ledeb.Fl.Alt. did not rely on summer rain, but groundwater instead (Xu and Li, 2006). This result is consistent with a report that the shallow-rooted species of *Nitraria sphaerocarpa* showed lower rainfall thresholds than that of deep-rooted species *Elaeagnus. angustifolia* (Zhao and Liu, 2010). According to a synthesis study in southern Australia, shrub and tree species’ transpiration response to rainfall events can be categorized into four modes: large and rapid; small and rapid; delayed, and no response depending upon plants’ discrepancy in plant water-use strategies (Burgess, 2006). Because of different adaptability for survival, species may not respond equally to the same rainfall events, and the rainfall thresholds and time lags also differ (Burgess, 2006; Zeppel et al., 2008; Zhao and Liu, 2010). For example, the stems and branches of the shrub species *Caragana korshinskii* Kom. and *Hippophae rhamnoides* Linn. on Loess Plateau showed different rainfall thresholds because of different plant water use type (Huang and Zhang, 2016; Jian et al., 2016).

Changes in rainfall pattern have been predicted by global climate models, with more frequent and intense rainfall events, particularly in water-limited regions (Peng et al., 2013). The Loess Plateau is located in the middle reaches of the Yellow River basin in northern China and is characterized by a semi-arid climate. A series of revegetation projects has been implemented in this area recently, which has led to changes in hydrological processes and soil erosion on the Loess Plateau (Zhao et al., 2017). *Vitex negundo* var. heterophylla (Franchet) and *Hippophae rhamnoides* Linn. are the dominant shrub species in this area and are used widely in ecological restoration (Wang et al., 2019; Yuan et al., 2022). However, the imbalance between water supply and demand is becoming particularly acute because of low rainfall and high atmospheric evaporative demands in this region (Jian et al., 2015). Studies have found a potential decreasing trend in the number of rainy days and increasing trends rainfall intensities and storm frequencies in Loess Plateau (Zhao et al., 2017; Zhao et al., 2018). Although the most recent studies have focused on *V. negundo* and *H. rhamnoides*’s water use and its influencing factors (Fang et al., 2019; Wang et al., 2019), little is known currently about the these two species’ water use strategies and the main influencing factors of SF responses to rainfall (Wu et al., 2018). Illustrating the individual plant’s responses to different rainfall magnitudes is critical to explore species’ adaptability to altered rainfall patterns. Rainfall events change the vapor pressure deficit (VPD, kPa), solar radiation (Rs, KW/m^2^), and the air’s relatively humidity (RH, %) as well as soil moisture. It is well accepted that SF variability is related to environmental factors, while SF’s key determining factors under different rainfall events in water-limited environments are still unclear (Wu et al., 2018; Liu et al., 2022). Although plant–water relations have been an important element of eco-physiological research, particularly in water-limited regions, previous studies have not quantified these factors’ effect under different rainfall amounts conditions.

Therefore, we conducted 3 years of field observations of the SF of two shrub species in this study, with the goal to identify the underlying determining factors and mechanisms in SF’s responses to different rainfall classes (defined by amounts) (Zhao and Liu, 2010; Huang and Zhang, 2016) on the semiarid Loess Plateau. The specific objectives of this study were to (1) investigate SF’s response to rainfall events and the primary controlling factors, and (2) explore the differences in the two species, i.e., *V. negundo* and *H. rhamnoides*’s water use strategies.

## 2 Materials and methods

### 2.1 Study area and plot design

**Figure 1:**
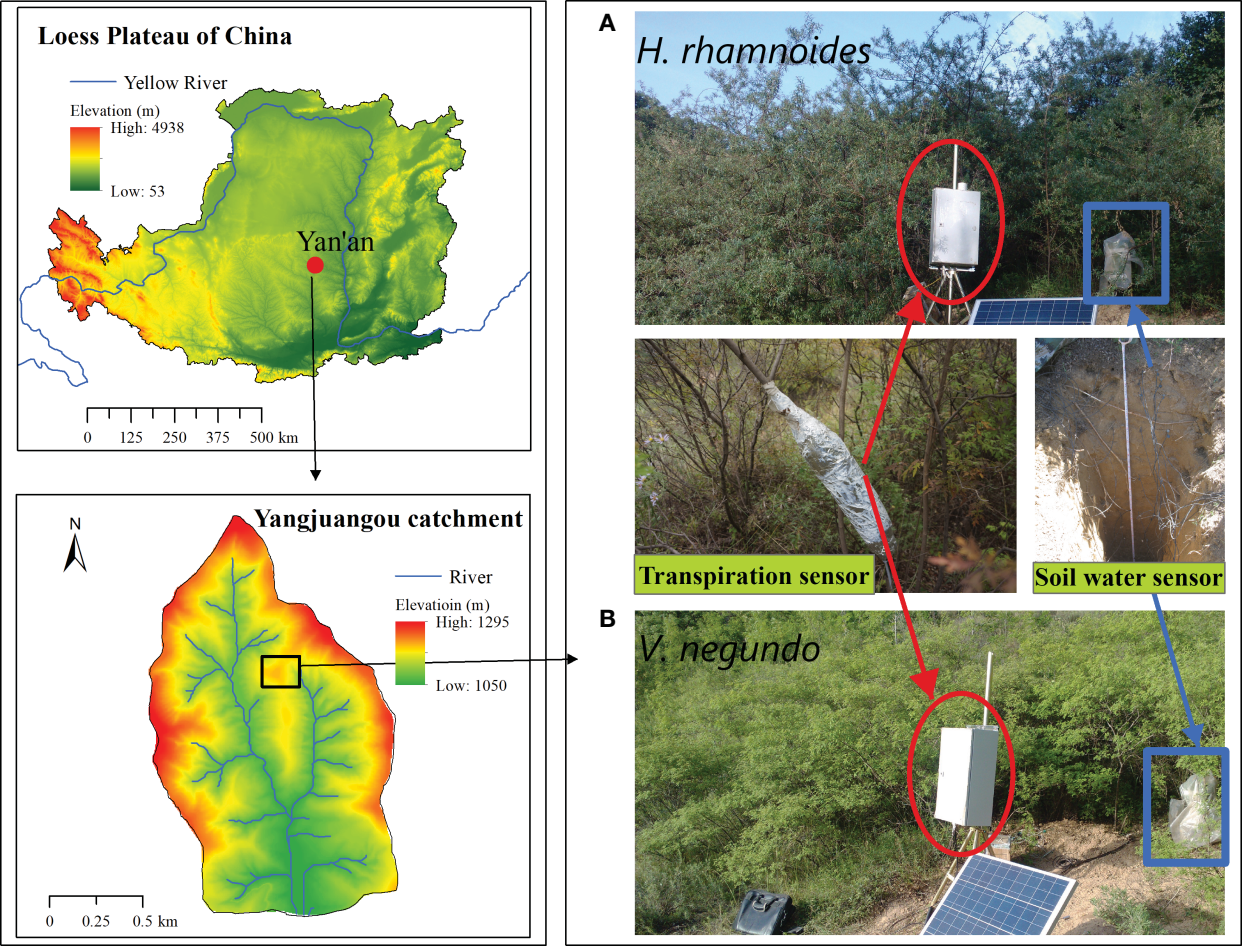
Location of the study area and experimental stands of *H. rhamnoides* **(A)** and *V. negundo* **(B)**, and example of heat balance gauge and soil moisture sensor.

The study site, the Yangjuangou catchment, is located in Yanan City, Shaanxi Province (36°42’ N, 109°31’ E), in the central part of the Loess Plateau (**Figure 1**). The climate is influenced primarily by the North China monsoon, with a dry winter and spring, a warm and comparatively wet summer, and a brief, cool autumn. The 60-year mean annual precipitation and air temperature are 530 mm and 10.6°C, respectively. Loessal soil is the main soil type in this catchment and the soil depth is usually 50–200 m depending upon the topography (Wang et al., 2012), with a high erosional sensitivity to water (Zhou et al., 2017). The groundwater is buried deeply and the soil water is the primary water source for plants (Chen et al., 2008; Fan et al., 2016). The natural vegetation is disturbed largely by long-term human activity.

Two typical shrub species, *V. negundo* and *H. rhamnoides*, were selected for this study. These two species are used widely in ecological restoration projects on the Loess Plateau because of their adaptability to semiarid conditions. *V. negundo* is a perennial deciduous, multi-stemmed shrub. A set of lateral roots is concentrated in the 60 cm soil layers and radiates out from the main root (Wang et al., 2017b). *H. rhamnoides* is a deciduous perennial shrub with long narrow leaves and a main root that extends downward to approximately 250 cm (Wang et al., 2019). Two 5×5 m plots that each of the two species dominated were established on the same hill slope with a middle slope position, aspect and slope degree between 10° and 15° to obtain similar micro-environmental conditions. The basic characteristics of the individual plants in the plots, such as height and stem diameter, were measured. The diameters and detailed information of the stems are shown in **Table 1**.

**Table 1:** Geographical parameters and biological parameters characteristic of two shrub stands of *V. negundo* and *H. rhamnoides*.

	Parameter	Mean SD	
		*V. negundo*	*H. rhamnoides*
Geographical parameters	Slope aspect	SW	SW
	Slope position	Middle	Middle
Biological parameters	Plant mean height (m)	1.54 ± 0.13 (2015)	1.65 ± 0.20 (2015)
		1.57 ± 0.09 (2016)	1.75 ± 0.21 (2016)
		1.58 ± 0.10 (2017)	1.82 ± 0.15 (2017)
	Mean base diameter (mm)	11.32 ± 0.28 (2015)	19.58 ± 0.94 (2015)
		12.88 ± 0.38 (2016)	23.08 ± 1.53 (2016)
		13.40 ± 0.51 (2017)	24.01 ± 1.32 (2017)
	Vegetation coverage (%)	62–73	54–60

### 2.2 Measurement of sap flow density and leaf water potential

Individual stems’ SF was measured using the heat balance method, which is adapted well to measure sap velocity in small-diameter stems (Yue et al., 2008; Jian et al., 2014). Previous studies have confirmed the accuracy of this technique (Dugas et al., 1993; Hall et al., 1998). The measurements were replicated with five to seven individuals of each species (**Table 2**). The details of installing the gauges can be found in previous studies (Fang et al., 2018; Wang et al., 2020). Samples stem basal diameter was measured for five to seven stems in each of the plots, and different types of sensors were used depending on that diameter (Dynamax Inc., Houston, TX, USA, Model SGA 13, SGB 19, and SGB 25) (**Table 2**). The stems selected for measurements were in good condition and developed sufficiently to support the sensors’ weight. The experiment was carried out from June to September in 2015 and 2017.

**Table 2:** Gauge types and diameters of the sample shrub used for the sap flow measurements.

Species	Gauge type (Wrapped stem area diameter cm^2^)							
*V. negundo*	2015	SGA 13 (1.87)	SGA 13 (1.95)	SGA 13 (2.31)	SGA 13 (1.56)	SGB 19 (2.16)	SGB 19 (3.27)	SGB 19 (4.24)
	2016	SGA 13 (1.79)	SGA 13 (1.59)	SGA 13 (1.82)	SGB 19 (3.82)	SGB 19 (2.85)		
	2017	SGA 13 (1.92)	SGA 13 (1.59)	SGA (2.40)	SGA (2.00)	SGB 19 (3.78)	SGB 19 (6.39)	
*H. rhamnoides*	2015	SGB 19 (4.98)	SGB 19 (4.10)	SGB 19 (3.55)	SGB 19 (4.38)	SGB 25 (8.03)	SGB 25 (7.53)	SGB 25 (7.35)
	2016	SGA 13(1.28)	SGB 19 (3.58)	SGB 19 (3.45)	SGB 25 (6.07)	SGB 25 (5.88)		
	2017	SGA 13(1.78)	SGA 13 (1.56)	SGB 19 (3.59)	SGB 19 (4.79)	SGB 25 (7.85)	SGB 25 (6.15)	

Leaf water potential (ψ_L_, MPa) was measured using a pressure chamber (PMS Instrument Co., Corvallis, OR, USA). The leaf blades were covered with a plastic bag before petiole excision and remained in the bag during measurement (Shellie and Bowen, 2014). Predawn ψ_L_ was measured 20 min before sunrise, and the daily ψ_L_ was measured every 2 hours from 0600 to 1800. Five replicate measurements were taken on small branches for each measurement; ψ_L_ was measured on June 16, and represented the pre-rainfall value, and on June 25, which was after a 14-mm rainfall event on June 23.

### 2.3 Measurements of environmental factors

An automated weather station was situated approximately 100 meters from the experimental field. All climatic data were measured once per minute and recorded every 30 minutes by a CR1000 data logger (Campbell Scientific Inc., Logan, UT) during the study period. The solar radiation (Rs, KW m^−2^) was measured using a pyranometer (LI-200, Li-Cor., Lincoln, NE, USA), which was installed approximately 2 meters above the ground in an open field. The rainfall amount was recorded using a tipping-bucket rain gauge (TE 525) mounted approximately 1 m above the ground. HMP35C probes (Vaisala Co., Helsinki, Finland) were used to measure air temperature (Ta, °C) and RH (%) with a data logger (Campbell Scientific, Logan, UT, USA) for an average of 30 minutes. The VPD was calculated based upon Ta and RH using the following equation:


$$\text{VPD=axexp}\left(\frac{\text{bxTa}}{\text{Ta+c}}\right)\times \left(1-RH\right)$$

Where Ta and RH were air temperature and relative humidity, constant term a=0.6111kPa, b=17.502, c=240.97°C (Campbell and Norman, 1998). The volumetric soil water content (SWC) was measured using eight EC-5 soil moisture probes for each plot (Decagon Devices Inc., Pullman, WA, USA) that were installed at eight depths below the soil surface (5, 10, 20, 40, 80, 120, 160, and 200 cm), and the data were recorded with a HOBO weather station logger (H21, Onset Computer Corp., Bourne, MA, USA) at 30-minute intervals.

### 2.4 Defining rainfall events

SF’s response to rainfall was evaluated by calculating its difference before and after a given rainfall events. “Before rain” refers to the day before one or several rain days and “after rain” refers to the day when the SF peaked after the same rainy day(s) (Zeppel et al., 2008; Zhao and Liu, 2010; Wang et al., 2017a). The response time lag refers to the time from after-rainfall days to the days when SF reached maximum values. Daily rainfall< 0.2 mm was considered rain-free because of the high VPD. The SF responses to rainfall events are also related to the antecedent soil moisture conditions (Reynolds et al., 2004). To comply with the characteristics of a rainfall event, the data were screened, and excluded the following situations: when rainfall events lasted longer than 5 days or the interval between the two rainfall events was less than 1 week (i.e., the logger could not distinguish the effects between two rainfall events) (Zeppel et al., 2008; Zhao and Liu, 2010). According to these criteria, 29 pairs of eligible rainfall events were identified during the study period from 2015 to 2017.

### 2.5 Data analysis

To investigate the SF’s response to microclimate, an integrated index referred to as the variable of transpiration (VT) was used to represent Rs and VPD. The VT was calculated as a simplified combination of Rs and VPD (VT=VPD×Rs^1/2^) (Du et al., 2011). Studies have shown that the relation between SF and VT can reflect the SF’s response patterns to atmospheric factors for each species under different soil water conditions after rainfall events (Du et al., 2011; Wu et al., 2018). The relation between SF and VT using the following exponential saturation function is as follows:


SF=a(1−exp(−bVT))

Where VT is the variable of SF, and a and b are fitting parameters.

The significance of the SF’s response to rainfall events was analyzed using one-way analysis of variance (ANOVA) to compare the main effects of rainfall and species (homogeneity of variance and normal distribution were tested). The *t*-tests were performed to examine the differences in environmental factors in the three main growing seasons (from June to September). Stepwise multiple linear regression analyses were conducted to identify the key factors that affecting SF for the two species. All statistical analyses were performed using SPSS v. 13.0 (SPSS Inc., USA), with *p* = 0.05 as the threshold for statistical significance. The explanation of environmental factors was identified according to the variation of regression coefficient. The positive or negative values of regression parameter for each factors indicated the positive or passive effect on SF. Graphic plotting was performed in Origin (Version 9.0, OriginLab Corp., USA). We used descriptive statistics to calculate means and standard error for each set of replicates. ANOVA and the least significant difference (LSD) were used to test for significance in the differences of contrasting treatments.

ψ_L_ against the corresponding SF values were plotted to obtain the apparent hydraulic conductance for two species under different water status conditions. The slope of the linear relation can be considered the plant’s leaf-specific apparent hydraulic conductance, and the intercept on the X axis (ψ_0_) is the water potential at the soil–root interface (Xu and Li, 2006). The consistency of leaf–specific conductance suggests that a balance between the soil–root system’s water supply and the atmosphere–shoot system’s water demand can be maintained (Xu and Li, 2006).

## 3 Results

### 3.1 Environmental variables in the study area

**Figure 2:**
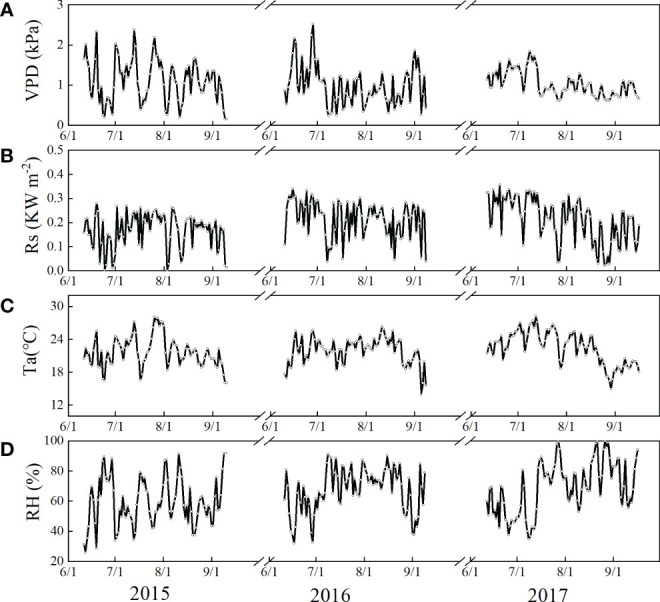
Variations in **(A)** mean daily vapor pressure deficit (VPD, kPa), **(B)** daily solar radiation (Rs, KW m^−2^), **(C)** mean air temperature (Ta, °C), and **(D)** relatively humidity (RH, %) in the study area from June to September during 2015–2017.

**Figure 3:**
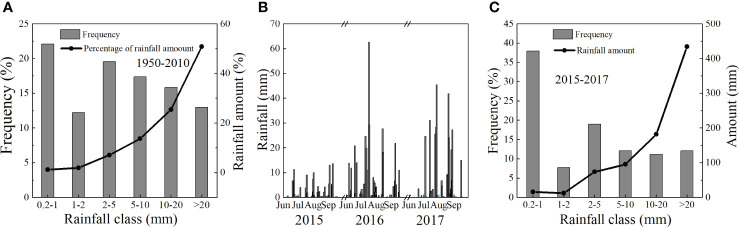
Frequency distribution and % of total rainfall amount as a function of rainfall classes from June to September during 1950–2014 **(A)**; frequency and rainfall amount **(B)**, and rainfall amount distribution **(C)** from June to September during 2015–2017.

Daily average VPD, Rs, and Ta displayed similar seasonal trends during June to September in 2015, 2016 and 2017, and the interannual variation in the tree did not significantly differ among the 3 years (*p* > 0.05) (**Figure 2**). Rainfall data collected from 1950–2010 indicated that the annual amount averaged 376.11 mm from June to September. The percentage of the total amount increased with increasing rainfall, while the frequency of events decreased. The trends during the experimental period was similar to that of the values from 1950–2010. Most rainfall events were 0.2–5 mm, which accounted for 64.66% of the total events, followed by 5–10 mm, > 20 mm, and 10–20 mm, which accounted for 12.93%, 12.07%, and 10.34%, respectively (**Figure 3**). Rainfall events greater than 10 mm were infrequent, but contributed highly to the total rainfall amount (74.53%).

**Figure 4:**
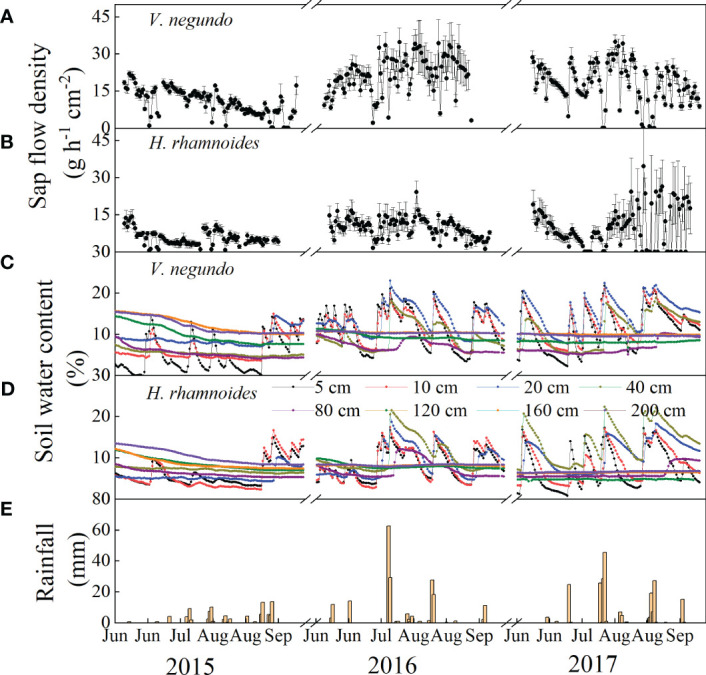
Variation of sap flow density and soil water content at different soil layer depths after selected rainfall events for the *V. negundo* **(A, C)** and *H. rhamnoides* **(B, D)** stands, and the 29 eligible rainfall events identified **(E)** during the study period from 2015 to 2017.

The SWC of *V. negundo* and *H. rhamnoides* communities in different soil layers exhibited different responses to rainfall events (**Figure 4**). The SWC in the upper soil layers (5–40 cm) depended highly upon rainfall patterns and had a high temporal variability.

### 3.2 Dynamic variation of SF with different rainfall classes

**Figure 5:**
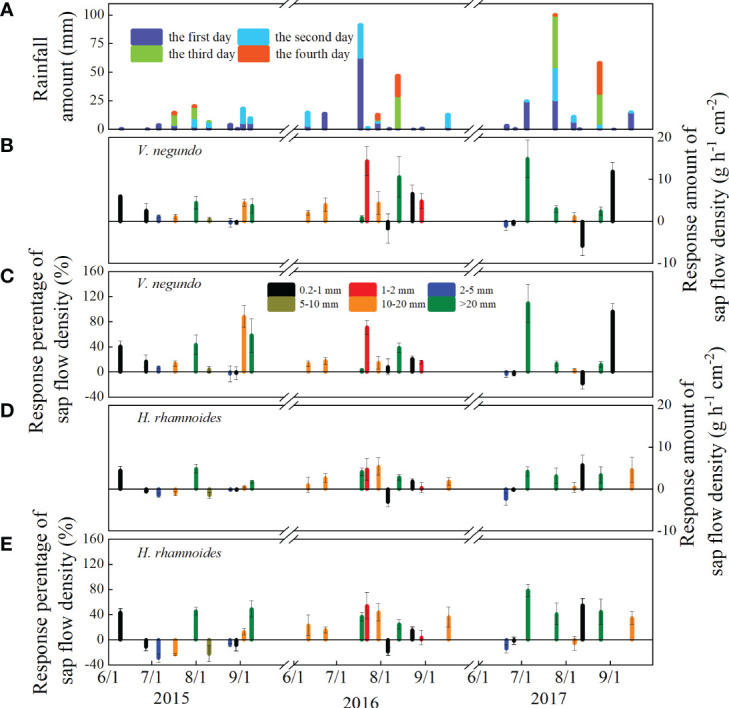
Different rainfall patterns **(A)** and dynamics of the amount and increased percentage of SF in *V. negundo* **(B, C)** and *H. rhamnoides* **(D, E)** from June to September during 2015–2017.

The diurnal courses of SF for these two species showed similar variation patterns from June to September (**Figures 4A, B**). In most cases, high SF values coincided with high SWC, Rs, and VPD. On days when rainfall occurred, SF values were much lower. The SF’s highest daily averages during the study period were 34.79 and 26.42 g h^−1^ cm^−2^, both of which occurred in August in 2017, and the smallest corresponding values were nearly zero on cloudy and rainy days for *V. negundo* and *H. rhamnoides*, respectively (**Figure 5A**). The increment of SF was between −5.88 and 14.38 g h^−1^ cm^−2^, −3.16 and 5.76 g h^−1^ cm^−2^ for *V. negundo* and *H. rhamnoides*, respectively (**Figures 5B, D**). The percentage of daily SF change in response to rainfall events ranged from −18.68 ± 8.86 to 109.82 ± 30.16% for *V. negundo* and −29.08 ± 6.73 to 78.67 ± 9.88% for *H. rhamnoides*, with mean values of 25.18 ± 6.36% and 18.40 ± 5.54% respectively. The largest increases in SF were approximately 109.82% and 78.67% after a 24.8 mm rainfall event in 4 Jul, 2017 for *V. negundo* and *H. rhamnoides*, respectively (**Figures 5C, E**).

**Figure 6:**
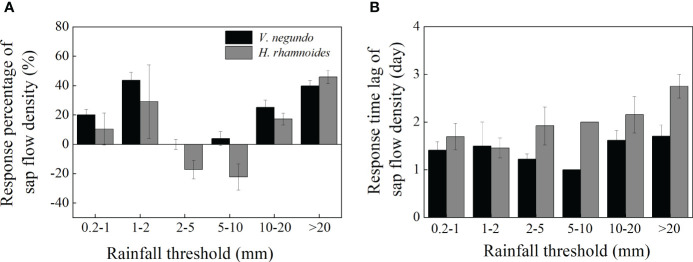
Percentage increase in the shrub species’ sap flow density (%) **(A)** and response time lag **(B)** for *V. negundo* and *H. rhamnoides*. Values represent the mean ± SD change in the increased percentage of SF from the first day before a rainfall event to the time when the maximum SF occurred after the rainfall event.

The two species’ SF responses differed among the rainfall amount classes. In general, SF increased significantly and decreased gradually first, and then increased as rainfall amounts increased. When rainfall events ranged from 0.2 to 1 mm and from 1 to 2 mm, the SF increased by an average of 19.97% and 43.49% for *V. negundo*, and 10.38% and 29.07% for *H. rhamnoides*, respectively (**Figure 6A**). In contrast, SF remained nearly stable for *V. negundo* after 2–5 mm rainfall events and increased by only 3.92% after 5–10 mm rainfall events, while it decreased by 17.30% and 22.40% after 2–5 mm and 5–10 mm rainfall events for *H. rhamnoides*. Conversely, SF increased gradually when rainfall events exceeded 10 mm. It increased by 25.10% and 39.84% for *V. negundo*, and by 17.25% and 45.80% for *H. rhamnoides* after 10–20 mm and above 20 mm rainfall events, respectively.

The lag times in the two species’ responses ranged from 1 to 3 days after rainfall, and *H. rhamnoides*’s response time was significantly longer than that of *V. negundo* (*p*=0.02). The lag times showed no significant differences among the rainfall classes for *V. negundo*, while the lag time at >20 rainfall class was higher than that at 0.2–2 mm for *H. rhamnoides* (*p*<0.01) (**Figure 6B**).

### 3.3 Relations between SF and environmental factors in different rainfall amounts

Multiple regression models for the relation between SF and environmental factors are shown in **Table 3**. In general, the explanations of meteorological factors and SWC were different among rainfall classes for SF variation for two species. When rainfall was less than 2 mm, the variation explained in Rs and VPD was 71%, while SWC accounted for only 8% of the variation in SF (**Table 3**). However, meteorological factors’ positive effect on SF decreased and the passive effect (RH) increased when rainfall ranged from 2 to 10 mm for both species. When the rainfall event was greater than 10 mm, the influencing factors of SF were Rs and Ta for the two species. For *V. negundo*, the variation in SF was correlated with SWC _0–40cm_ when rainfall was less than 10 mm, while *H. rhamnoides*’s variation in SF was more sensitive to SWC_40–120cm_ for the three classes (**Table 3**).

**Table 3:** Multiple linear regressions between SF and environmental factors after different rainfall classes.

Species	Rainfall classes	Regression equation	Variation explained			
			Total	Meteorological		SWC
				positive	passive	
*V. negundo*	0.2−2	SF=74.98Rs+1.84SWC_0−40cm_+9.57VPD−2.23SWC_120−200cm_+0.89	79%	71%		8%
	2−10	SF=38.02Rs+12.02SWC_0−40cm_+1.39SWC_120−200cm_−0.39RH−5.59VPD−66.85	79%	52%	14%	13%
	>10	SF=61.81Rs+1.60Ta+4.88SWC_40−120cm_−0.49RH−3.92SWC_120−200cm_+8.17	81%	58%	2%	21%
	All	SF=59.71Rs+1.36Ta+5.55SWC_40−120cm_−3.20SWC_120−200cm_−0.53RH+6.08	71%	59%	4%	8%
*H. rhamnoides*	0.2−2	SF=32.53Rs+1.08SWC_40−120cm_+1.67VPD−8.11	80%	70%		10%
	2−10	SF=16.46Rs+2.39SWC_40−120cm_−0.33RH−5.70VPD+46.15	73%	48%	15%	10%
	>10	SF=29.05Rs+2.32SWC_40−120cm_+1.02SWC_120−200cm_−0.22RH+6.54	72%	53%	5%	14%
	All	SF=28.44Rs+1.88SWC_40−120cm_−0.21RH−0.60SWC_120−200cm_−3.00VPD+7.87	68%	55%	2%	11%

**Figure 7:**
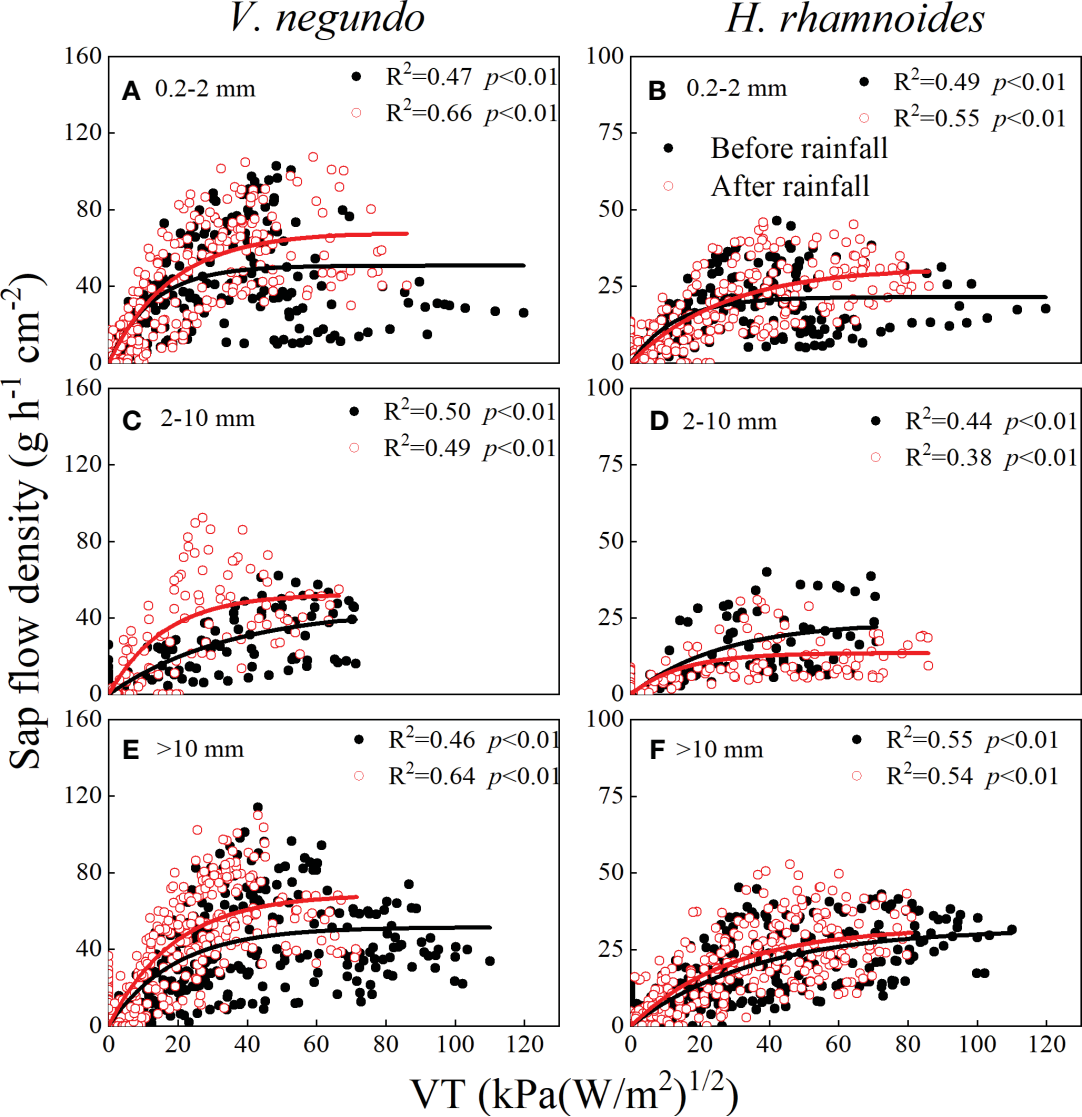
Relationships between SF and variable of transpiration (VT) of *V. negundo* **(A, C, E)** and *H. rhamnoides* **(B, D, F)** during each pair of rainfall events.

To elucidate SF’s response patterns to atmospheric factors, SF and VT values were analyzed before and after different rainfall classes for the two species (**Figure 7**). Generally, SF increased in response to rising VT, while the regression coefficient differed before and after rainfall periods for *V. negundo* when the rainfall amount was 0.2–2 and above 10 mm. *V. negundo*’s average regression coefficient was nearly 1.4 times and greater after rainfall than that of pre-rainfall. In contrast, the regression coefficient was nearly equal for *H. rhamnoides* before and after the three rainfall classes, suggesting less amplitude in SF’s response of to changes in atmospheric factors.

### 3.4 Variation of leaf water potential and leaf-specific hydraulic conductance to rainfall event

**Figure 8:**
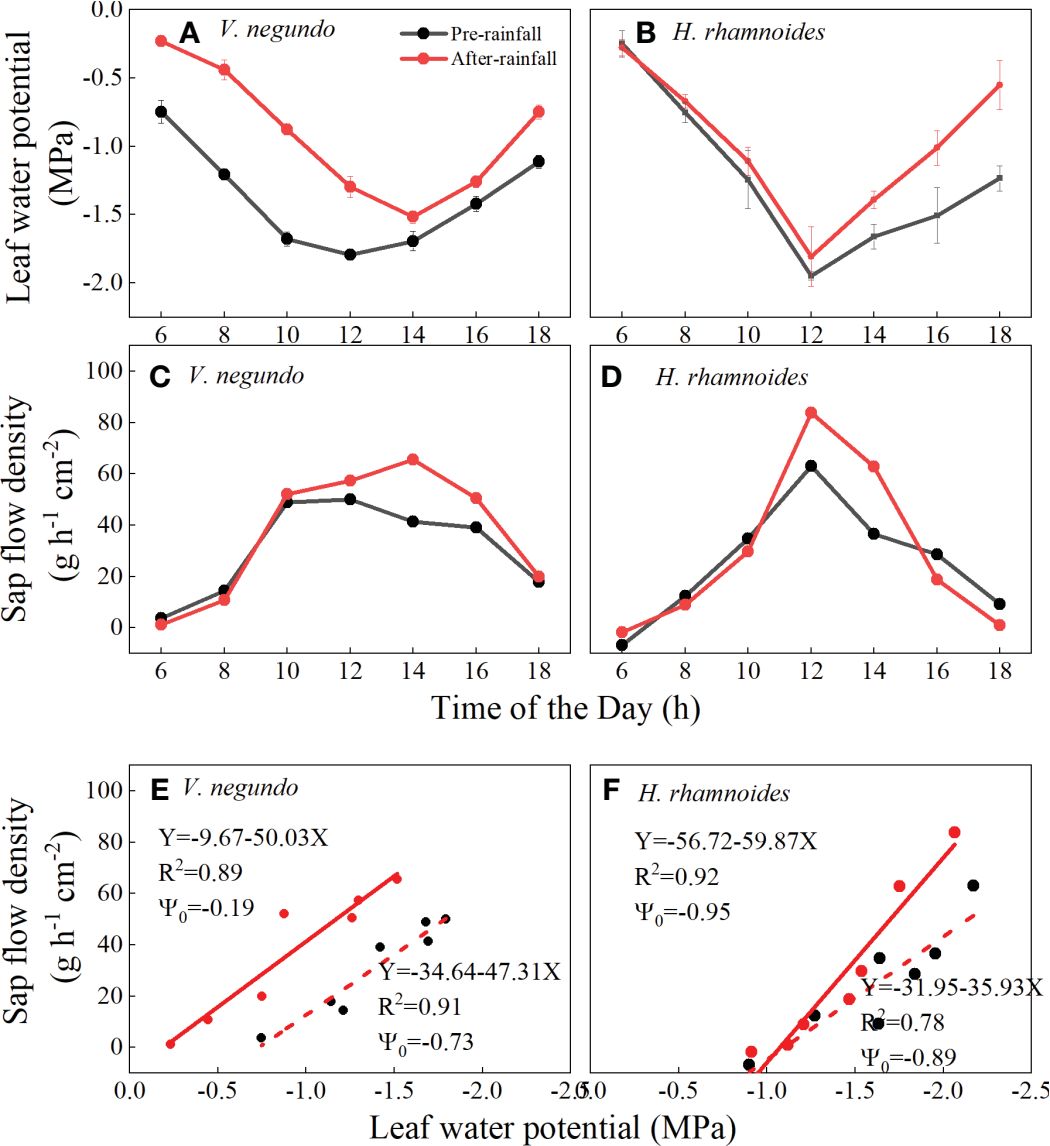
Diurnal variation in leaf water potential **(A, B)**, sap flow density **(C, D)** and leaf-specific apparent hydraulic conductance **(E, F)** of *V. negundo* and *H. rhamnoides* before and after a 14 mm rainfall event.

The daytime courses of ψ_L_ in *V. negundo* increased significantly after a 14 mm rainfall event (*p*<0.01), while that of *H. rhamnoides* remained at nearly similar levels before midday and did not change significantly after rainfall (*p*>0.05) (**Figures 8A, B**). The ψ_L_’s minimum value and SF’s maximum value occurred at 1400 and 1200 after rainfall for *V. negundo* and *H. rhamnoides*, respectively (**Figures 8C, D**).

The two species’ leaf-specific hydraulic conductance and ψ_0_ behaved differently: the leaf-specific hydraulic conductance remained relatively stable under different soil water statuses in the upper layer for *V. negundo* (**Figure 8E**), and increased for *H. rhamnoides* (**Figure 8F**). The ψ_0_ was −0.19 and −0.73 MPa for *V. negundo* before and after rainfall, respectively, while it was ∼−0.90 MPa for *H. rhamnoides* before and after rainfall.

## 4 Discussion

As the primary source of water replenishment in water-limited regions, rainfall plays an important role in sustaining vegetation physiological processes (Weltzin and Tissue, 2003). An increased SF could trigger a cascade of responses that affect plant growth and survival (Wang et al., 2020; Iqbal et al., 2021; Liu et al., 2022), particularly in water-limited areas. The differences in the species’ biological characteristics and water use strategies affect their sensitive responses to environmental factors (Xu and Li, 2006; Wu et al., 2018).

### 4.1 Sap flow’s response to rainfall events

The SF’s response to rainfall events is related to multiple environmental controlling factors and physiological characteristics (Zeppel et al., 2008; Wang et al., 2017a). There existed differences in the fitting parameters from the multiple regression models during before and after rainfall for two species, suggesting that the transpiration process is sensitive to changing environmental conditions, although there was a discrepancy in the key influencing factors on SF under different rainfall amounts. Small rainfall events wet the plant surface, and plants can absorb rainwater that adheres to their leaves and stems (through lenticels) and use the water to increase SF (Zhao and Liu, 2010; Yuan et al., 2017). The SF’s percentage response was larger for *V. negundo* than for *H. rhamnoides* after small rainfall events (0.2–2 mm) in this study (**Figure 6**), possibly because of *V. negundo*’s the larger and coarser leaf and bark texture, which may enhance the adsorption capacity effectively. Studies also have shown that the effect of Rs and RH in increasing SF is as important as that of rainfall itself (Zhao and Liu, 2010; Wang et al., 2017a). The effects of meteorological factors on transpiration were greater for *V. negundo* than for *H. rhamnoides* (Fang et al., 2019), and therefore, SF’s the response to rainfall amounts was lower for *H. rhamnoides* than that of *V. negundo*.

Rainfall increased the air’s RH and decreased the evaporative demand when rainfall was higher than the upper threshold for a species (Huang and Zhang, 2016), and the effect of meteorological factors result in a decrease in SF. The relative increase in transpiration for a Scots pine forest in the Bruntland Burn catchment decreased as the amounts of rainfall increased because the response of transpiration to environmental factors decreased (Wang et al., 2017a). In this study, when the rainfall amount was 2–10 mm, the effect of RH was greater than that of other classes. Therefore, the lower atmospheric evaporative demand limited the variation in SF.

The rapid increase in SF after rainfall events greater than 10 mm for *V. negundo* and *H. rhamnoides* could be attributable to the combination of meteorological factors and increased soil moisture. Large rainfall amount led to a sharp increase in soil moisture and maintained a significant soil moisture response (**Figures 4C, D**), which led to a rapid increase in SF. This reflected soil moisture content’s effects on the roots’ ability to supply water to the canopy and the atmosphere to drive evaporation from the canopy (Zeppel et al., 2008; Huang and Zhang, 2016). Soil water influences SF through the fluxes of water within the root zone. Thus, root depth can also affect plant–water relations following a rainfall event (Ogle and Reynolds, 2004; Burgess, 2006). In this study, the roots were distributed primarily in the upper layer, with 79% and 62% within 0–100 cm, and the maximum root depth was 200 cm and 250 cm for *V. negundo* and *H. rhamnoides*, respectively (Wang et al., 2017b; Wang et al., 2019). Superficial roots mass affected transpiration’s response to small rainfall events (2 mm) (Burgess, 2006), so the increased percentage of SF was higher when rainfall less than 2 mm. Studies have shown that *V. negundo* nearly used the upper soil profile moisture (0–40 cm), while the *H. rhamnoides* absorbed a higher percentage of deep soil moisture (120–300 cm) (Wang et al., 2017b; Wang et al., 2019). Therefore, *H. rhamnoides*’s response time lag increased slightly with an increase in rainfall amount and was greater than that of *V. negundo*. Because of the difference in root depths, the lower rainfall thresholds of stems’ response to rainfall occurred at 4.8 and 5.2 mm for *N. sphaerocarpa* and *E. angustifolia*, respectively (Zhao and Liu, 2010). In addition to root depth, the response of SF to soil moisture also was affected by the antecedent soil water conditions (Engel et al., 2005). For example, the SF response was lower after a 91.8 mm rainfall on 17 July in 2016 that that value after a 29 July in 2016, because that the antecedent water availability was higher owing to consecutive rainfall of 30.6 mm rainfall from 8 to 12 July in 2016.

Structure determines function (Yuan et al., 2022), and fine roots are an important active physiological component in water uptake (Schenk, 2008). This result is similar to that of the creosote bush (*Larrea tridentata*), which assimilates soil nitrogen into the leaves within 2 days after 20 mm of rainfall (BassiriRad et al., 1999); thus, soil moisture and nutrients are available to plants more readily after an effective rainfall event for soil moisture, which enhances the plants’ ability to respond to light conditions (i.e., begin transpiration) (Zhao and Liu, 2010).

### 4.2 Types of water use strategies between the two species

The daytime pattern of SF and ψ_L_ is related to the species’ water use strategies (Du et al., 2011), and the plants responded to rainfall events by regulating their water potential which influenced the hydraulic conductance (Zhao and Liu, 2010). The variation in ψ_L_ after rainfall indicated that the two species had different physiological regulation after rainfall (Xu and Li, 2006). Although SF’s response showed a similar trend to the rainfall amount for the two species, the variation in ψ_L_ indicated different water use strategies on the part of the two species. In this study, *H. rhamnoides* had a narrow range of ψ_L_ before and after rainfall, which was similar to that Jian et al. (2016) reported. Thus, *H. rhamnoides* tended to be a isohydric species, which is attributed generally to strong stomatal control of transpiration, while a relatively constant ψ_L_ is maintained (West et al., 2008; Fang et al., 2018). The significantly increased ψ_L_ after rainfall indicated that *V. negundo* can be considered an anisohydric species, which exhibits less stomatal sensitively to soil moisture and allows large fluctuations in ψ_L_. In addition, *V. negundo*’s increased ψ_0_ after rain indicates that the root system reacted to improved soil water potential after rainfall events. Wu et al. (2018) suggested that *H. rhamnoides* should be considered an anisohydric species because of fluctuations in ψ_L_. In general, plant water use types were continuum, rather than a dichotomy in isohydric and anisohydric behaviours (Klein, 2014). Even the same species could shift from isohydric to anisohydric behaviours under different soil moisture (Conesa et al., 2016). Against the background of climate change, precipitation appears to shift toward fewer and more intense rainfall events in water-limited regions (Hafner et al., 2020; Liu et al., 2022). The water use characteristics and mechanisms in environmental stress need further investigation to understand revegetation species’ adaptation strategies and long-term hydrological regime.

### 4.3 Limitations of this study

This study had certain limitations. First, the SWC measurement was based upon one soil moisture probe in each soil layer. Second, the variation in ψ_L_ was based upon one rainfall event. Third, the antecedent soil moisture conditions were not analyzed in this study. Longer experiments over a larger area should be conducted to examine revegetation species’ rainfall adaptation under climate change more holistically.

## 5 Conclusions

This study discussed the effects of rainfall events on *V. negundo* and *H. rhamnoides*, the main revegetation shrub species on the semiarid Loess Plateau of China. The results showed differences in the species’ SF and time lags in response to different rainfall amounts. The magnitude of the responses to different amounts depended upon the biological characteristics and water use strategy, which has shallower roots and a coarser leaf and bark texture, responded to small rainfall amounts by regulating its water potential at the soil–root interface and leaf water potential. *H. rhamnoides*, which is deep-rooted and has a leathery leaf, responded to larger rainfall amounts. The variation in leaf water potential after rainfall suggested that *V. negundo* exhibits anisohydric characteristics, while *H. rhamnoides* is an isohydric species, which can tolerate long-term, intense drought conditions. This study provides valuable information for screening species suitable for revegetation and ecological management, and the plant water use characteristics in the context of rainfall pattern changes require further investigation to understand the long-term adaptation of the species on the semiarid Loess Plateau region.

## Data availability statement

The original contributions presented in the study are included in the article/supplementary material. Further inquiries can be directed to the corresponding authors.

## Author contributions

WF: Conceptualization, Methodology, Investigation, Data curation, Formal analysis, Writing – original draft, Funding acquisition. NL: Investigation, Supervision, Writing – review & editing, Funding acquisition. JL: Investigation, Data curation, Writing – original draft, review & editing. RL: Data curation, Writing – review & editing. YW: Data curation, Supervision, Writing – review & editing. All authors contributed to the article and approved the submitted version.
